# Electrorheological Effect of Suspensions of Polyaniline Nanoparticles with Different Morphologies

**DOI:** 10.3390/polym15234568

**Published:** 2023-11-29

**Authors:** Jinhua Yuan, Xufeng Hu, Xiaopeng Zhao, Jianbo Yin

**Affiliations:** Smart Materials Laboratory, Department of Applied Physics, Northwestern Polytechnical University, Xi’an 710129, China; yuanjinhua@mail.nwpu.edu.cn (J.Y.); h-xf@mail.nwpu.edu.cn (X.H.); xpzhao@nwpu.edu.cn (X.Z.)

**Keywords:** electrorheological effect, particle morphology, rheological test, molecular dynamics simulation

## Abstract

Polyaniline (PANI) nanospheres, nanofibers, and nanoplates were prepared using the oxidative polymerization method. Scanning electron microscopy (SEM) was used to observe the three morphologies of PANI, and their structure was tested using infrared spectroscopy, X-ray diffraction, Raman spectroscopy, and X-ray photoelectron spectroscopy. The influence of particle morphology on the electrorheological (ER) effect was studied through rheological experiments and molecular dynamics (MD) simulation. The experimental and simulation results indicate that without applying an electric field, the nanofibers easily form a three-dimensional network structure in the suspension, resulting in yield stress. The three-dimensional network structure of the nanoplate suspension becomes weaker and the PANI nanosphere suspension lacks the ability to form a three-dimensional network structure. After applying an electric field, under the same condition, the yield stress and electric field-induced shear stress increment of PANI nanofibers are the highest, followed by nanoplates, and those of PANI nanospheres are the lowest. This indicates that the ER effect increases with the increase in particle morphology anisotropy. Through three-dimensional visual simulation analysis, it can be concluded that the enhanced ER effect associated with increased particle anisotropy can be attributed to improved stability in the ER chain structure.

## 1. Introduction

Electrorheological (ER) fluid is an intelligent suspension, consisting of dispersed-phase particles and dispersing media that can continuously and quickly regulate its rheology behavior with an external electric field [1,2]. It holds promising potential for various applications, including dampers, biomorphic robotics, microfluidic fields, etc. [3,4,5]. However, the present ER fluid has a low electric field-induced ER effect, which restricts its practical applications.

An in-depth understanding of the correlation between the structure and ER effect is crucial for preparing high-performance ER fluids. Particularly in recent years, researchers have conducted extensive studies about the impact of chemical and physical properties of ER particles on ER effect. Among these, particular attention has been given to exploring the particle morphology effect [6,7], such as sphere-like [8], core–shell-like [9,10,11,12], rod-like [13], plate-like [14], bowl-like [15], etc. [16]. Asano et al. reported one of the earliest experiments about utilizing elongated rod-shaped particles as ER particles [17,18]. The ER effect of suspensions containing microfibers made from poly(phenylene benzobisthiazole) was also investigated by Kanu and Shaw. They observed that as the aspect ratio increased, the ER effect increased [19]. The reason for the increase in ER effect can be put down to the large long-axis polarization and interaction force of anisotropic particles. Otsubo also examined the impact of particle morphology on the ER effect by comparing a whisker-like aluminum borate suspension with a spherical aluminum borate suspension [20,21]. The whisker suspension exhibited a significantly higher ER effect, as evidenced by both shear viscosity at steady and oscillatory viscoelastic tests. It has been considered that the increase in the ER effect caused by anisotropic morphology is primarily attributed to the formation of an intricate network structure composed of elongated particles. However, the studies above mainly focused on micro-size particles. In 2006, we developed a titanate nanofiber ER suspension, which also has an enhanced ER effect [22]. Since then, nanofiber ER fluids with an anisotropic structure have attracted a lot of attention [23,24,25,26,27,28,29,30,31,32,33,34]. For example, in 2007, Lin et al. found that a carbon nanotube/isopropanol suspension had a stronger ER effect compared to a spherical carbon suspension [23]. The ER effect of a polyaniline (PANI) nanofiber suspension, prepared in 2008, was found to be significantly higher compared to that of a granular PANI suspension [24]. In 2010, Cheng et al. developed a polar molecule-containing titanium oxalate nanofiber suspension showing a better ER effect than a polar molecule-containing titanium oxalate nanoparticle suspension [27]. In addition, various composite nanofibers or nanorods were also developed for ER suspensions [28,29,30,31,32,33,34]. The enhanced ER effect was also found in the elongated nanoparticles.

Besides nanofibers, nanoplates have also been studied as the dispersed phase of ER fluid [35,36,37,38,39,40,41,42]. For instance, Wang et al. synthesized chitosan-modified graphene nanosheets and found they exhibited an ER effect under an electric field [35]. Choi et al. prepared graphene oxide/PANI nanosheets and also found that they exhibited a typical ER effect [36]. Jang et al. proposed a straightforward solvent exchange method for the production of graphene oxide (GO) sheet suspension and found a higher ER effect than mechanically milled GO sheet suspension [37]. We prepared PANI-decorated graphene nanosheets and observed a remarkable improvement in the ER effect compared to granular PANI [38]. We also reported ER-suspension silica-coated graphene nanosheets [39]. The incorporation of a silica coating effectively mitigates the high electrical conductivity of graphene, leading to a significant ER effect under an alternating current (AC) electric field. Despite the demonstrated enhancement of the ER effect by the elongated morphology of dispersed ER nanoparticles, a systematic comparison of the effect of particle morphology on the ER effect is still lacking. This may be because of the preparation difficulty of ER nanoparticles with the same chemical and physical properties but different morphologies.

On the other hand, despite taking into account the impact of nanoparticle morphology on the ER effect, comprehending the impact mechanism remains challenging due to the nanoscale nature of particles and difficulties in accurately observing their structure under electric and shear fields. Consequently, simulating both the ER structure and its ER effect has emerged as a crucial approach [43,44,45,46,47,48,49,50]. Blair et al. used the Monte Carlo method to simulate and study spherical and non-spherical ER particle suspensions [45]. The simulated results demonstrated that as the electric field strength increased, the chain formation of rod-shaped nanoparticles following the specified direction of the electric field became more obvious compared to sphere-shaped particles. See et al. proposed a particle scale simulation model to study the impact of spindly nanoparticles on the micromorphology and field-evoked flow reaction of an ER suspension [46]. The simulated results showed that the system containing elongated particles produced a stronger ER effect compared with spherical particles. However, there is also a lack of systematic comparisons of the effect of nanoparticle morphology on the ER effect in the simulation studies.

In this study, we prepared three different morphologies of PANI nanoparticles, namely nanospheres, nanofibers, and nanoplates. We then conducted comprehensive research about the impact of particle morphology on the ER effect using rheological tests and molecular dynamics (MD) simulation. These three kinds of PANI particles have identical chemical structures and electric properties. The relevant ER effect was evaluated and compared at an equivalent volume fraction. Subsequently, we used the molecular dynamics-stochastic rotation dynamics (MD-SRD) method to develop models for three PANI ER suspensions and simulated their rheological behavior. Finally, the impact of nanoparticle morphology on the ER effect was discussed on the basis of experimental and simulated findings.

## 2. Experimental Section

### 2.1. Materials

Aniline (99.5%) and citric acid (CA, 99%) were purchased from China National Pharmaceutical Group Chemical Reagent Co., Ltd. (Shanghai, China). Before use, aniline needed to undergo vacuum distillation to remove oxides. Ammonium persulfate (APS, 98%) was purchased from Tianjin Tianli Chemical Reagent Co., Ltd. (Tianjin, China). Dimethyl silicone oil (KF-96, 50 cSt, 0.96 g/cm^3^ at 25 °C) was purchased from Shin-Etsu Chemical Co., Ltd. (Tokyo, Japan). The ultra-pure water used was self-made in the laboratory (Elco Analytical Ultra-Pure Water Machine, KL-UP-II-10 Type).

### 2.2. Preparation of PANI Nanoparticles with Different Morphologies

Preparation of PANI nanospheres. Initially, we dissolved 11.4 g of aniline and 25 g of citric acid in 600 milliliters of distilled water, ensuring that the solution’s pH was ≤2. Then, while stirring at 1200 rpm, we added an aqueous solution containing 27.4 g of APS to the aniline mixture. Finally, the solution was reacted in an ice bath (~4 °C) for 24 h without agitation to obtain a dark green PANI nanosphere suspension. The yield was 90%.

Preparation of PANI nanofibers. Initially, we dissolved 11.3 g of aniline and 8 g of citric acid in 600 milliliters of distilled water, ensuring that the solution’s pH = 3. Then, while stirring at 2500 rpm for 30 s, we swiftly added an aqueous solution containing 27.4 g of APS to the aforementioned aniline solution. Finally, the solution was reacted in an ice bath (~4 °C) for 24 h without agitation to obtain a dark green PANI nanofiber suspension. The yield was 87%.

Preparation of PANI nanoplates. Initially, we added 144 milligrams of GO and 50 milliliters of ethanol into a 1 M HClO_4_ aqueous solution of 120 milliliters and treated it with ultrasound for 30 min until the GO was completely dispersed. Next, under stirring at −10 °C, 1.6 g of aniline was added to form a yellow suspension containing GO and aniline. After stirring for another 30 min, a solution of 1.32 g of APS in 40 milliliters of 1 M HClO_4_ was added to the GO/aniline suspension. The resulting mixture was stirred at −10 °C for about 20 h to produce an emerald precipitate. Finally, the precipitate was filtered and washed with copious amounts of water and ethanol, and the dark green PANI nanoplates were obtained after drying at 80 °C. The yield was 80%.

Dedoping treatment of PANI particles. The PANI particles obtained via the above oxidative polymerization in an acid environment are emeraldine salt conductors. To prepare ER suspensions, the PANI was treated in an ammonia solution to form a semiconducting PANI emeraldine base. In order to ensure a fair comparison, the conductivity of the three samples was adjusted to the same level by regulating the dedoping time. Specifically, 3.0 g of the aforementioned PANI nanospheres was dispersed into 100 milliliters of 1 M ammonia solution and stirred at 25 °C for 2 h to obtain PANI base nanospheres with an electrical conductivity of 5.6 × 10^−10^ S/cm. Similarly, by dispersing 3.0 g of the above PANI nanofibers into 100 milliliters of a 1 M ammonia solution and stirring at 25 °C for 10 h, we obtained PANI base nanofibers with an electrical conductivity of 6.2 × 10^−10^ S/cm. Likewise, by dispersing 3.0 g of the above PANI nanoplates into 100 milliliters of a 1 M ammonia solution and stirring at 25 °C for 10 min, we obtained PANI base nanoplates with an electrical conductivity of 4.5 × 10^−10^ S/cm.

### 2.3. ER Suspension Preparation

The dried PANI particles with various morphologies were dispersed in methyl silicone oil (dielectric constant 2.7~2.9, dynamic viscosity 50 cSt, density 0.96 g/cm^3^ at 25 °C) to form an ER suspension with 10 vol%.

### 2.4. Characterization and Measurements

The particles were observed using a scanning electron microscope (SEM, JSM-6700, JEOL Co., Ltd., Tokyo, Japan) to determine their morphology. The samples were analyzed via Fourier transform infrared spectroscopy (FT-IR, JASCO FT/IR-470 plus, JASCO INTERNATIONAL Co., Ltd., Tokyo, Japan) to identify their chemical groups. The wavenumber range used for analysis was 400–4000 cm^−1^ with a resolution of 4 cm^−1^. The Raman spectra were measured using a Micro-Raman spectrometer (Raman, Invia, Alpha 300R, WITec Co., Ltd., Ulm, Germany) at 532 nm. X-ray photoelectron spectroscopy (XPS, Thermo Scientific K-Alpha, Waltham, MA, USA) was performed with a monochromatic AlKa source. All the XPS spectra were corrected using the C 1s line at 284.6 eV. Conductivity measurements were performed on pellets compressed from powders (20 MPa) at room temperature using a digital high ohmmeter (Keithley 6517B, Tektronix Inc., Portland (Beaverton), OR, USA). The dielectric spectra of ER suspensions were tested using a dielectric analyzer (HP 4284A, Agilent Technologies Inc., Palo Alto, Santa Clara, CA, USA) at room temperature.

The rheological properties of ER suspensions were tested with an electro-rheometer (Thermal-HAAKE RS600, HAAKE Inc., Karlsruhe, Germany) at 298 K. The test fixture was a PP ER 30 parallel plate system, while the high-voltage direct current (DC) required was generated using a high-voltage differential amplifier generator (10 kV, 2 mA, WYZ-010, Beijing, China). A computer-controlled circulating oil bath system (Phoenix) was used to control the temperature. The rheological curves were determined by controlling the shear rate from 0.1 s^−1^ to 1000 s^−1^.

## 3. Simulation Section

### 3.1. Models

**Figure 1:**
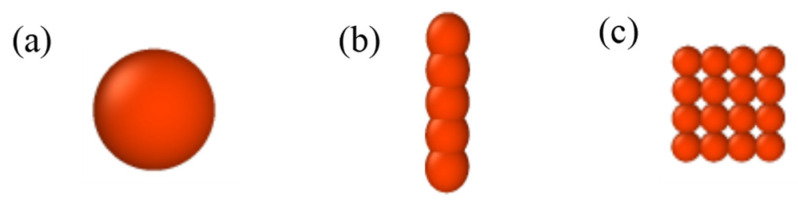
Different morphologies of PANI particles models: (**a**) nanosphere; (**b**) nanofiber; (**c**) nanoplate.

**Figure 2:**
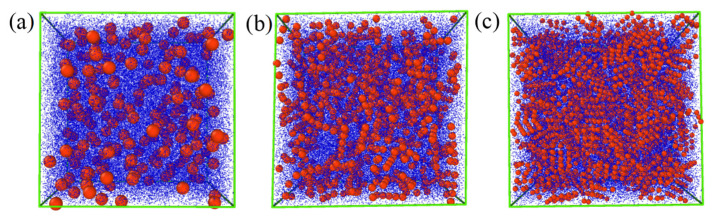
Different morphologies of PANI ER suspension models: (**a**) nanosphere; (**b**) nanofiber; (**c**) nanoplate. Blue particles represent silicone oil (dispersing medium SRD particles) and red particles represent PANI nanoparticles (dispersed-phase particles).

In the PANI ER suspensions, the PANI nanoparticles are the dispersed particle phase, and silicone oils the dispersing medium. Firstly, the models of three kinds of PANI nanoparticles with different morphologies are established. Figure 1a shows the PANI nanospheres model. Figure 1b shows the PANI nanofiber model. Note that the nanofiber is composed of five small balls with the same volume. There is a strong force between the small balls and the nanofiber can be regarded as a rigid body. Figure 1c shows the PANI nanoplate model. The nanoplate is composed of 16 small balls with the same volume. There is a strong force between the small balls, and the nanoplate can be regarded as a rigid body. Silicone oil is considered to be composed of *N* coarse-grained particles, which are called matrix particles. Then, the PANI ER suspension model is established by placing a specific volume fraction of PANI nanoparticles and matrix particles in the simulation box about *L* on side length, as depicted in Figure 2.

**Figure 3:**
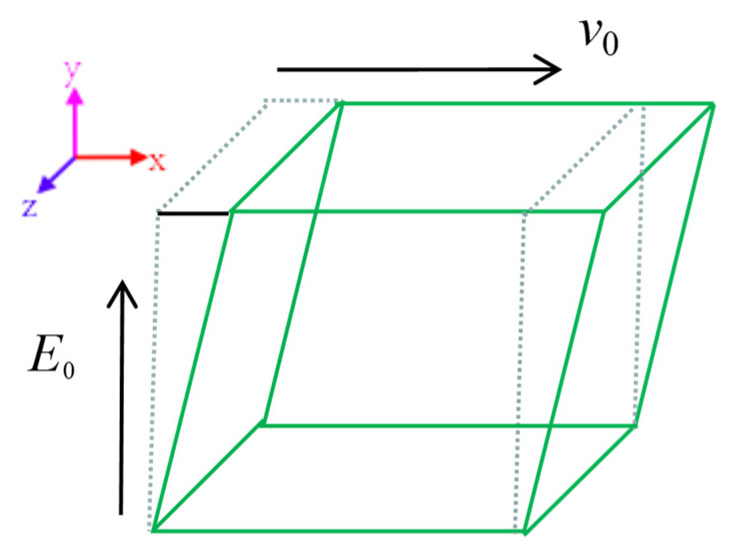
The depiction of the electric field and shear field.

The bottom of the box in Figure 3 is fixed, and the top can move at a steady speed $v_{0}$ in the *x* direction. Because of the box with a small side length, the velocity distribution of the suspensions between the bottom and top boundaries is approximately linear and there is a simple stable-state shear field with a velocity gradient of dv/dy. As shown in Figure 3, the dashed line represents the initial structure of the calculation box, while the solid line represents the structure after shear deformation of the calculation box. The shear gradient is in the *y* direction, the applied electric field is in the *y* direction, and there is no external load in the *z* direction. Therefore, after comprehensive consideration, the *z* direction and *x* direction are set as periodic boundary conditions (PBC), and the *y* direction is set as the Lees–Edwards boundary condition (LEBC), combined with other commands in LAMMPS, such as fix deform and fix nvt/sllod, etc.

#### 3.1.1. Electric Dipole Moment of Nanospheres

There are 191 nanospheres in the simulation and the nanospheres have a total volume fraction of 10 vol%, and the diameter of a nanosphere is $d=10^{-6}\;m$. Simultaneously applying electric and shear fields, this dipole moment changes and the final result is as follows [51,52]:

(1) $$\vec{p}=\frac{p_{0}}{1+{\left(\Omega \lambda \right)}^{2}}{\vec{e}}_{y}+\frac{\Omega \lambda p_{0}}{1+{\left(\Omega \lambda \right)}^{2}}{\vec{e}}_{x}$$

where

(2) $${\vec{p}}_{0}=\frac{1}{2}\pi \varepsilon_{0}\varepsilon_{f}d^{3}\beta_{\mathrm{eff}}{\vec{E}}_{0}$$

(3) $$\beta_{\mathrm{eff}}^{2}=\beta_{d}^{2}\frac{[{\left(\Omega \lambda \right)}^{2}+{\left(\frac{\beta_{c}}{\beta_{d}})\right]}^{2}+{\left(\Omega \lambda \right)}^{2}{\left[1-(\frac{\beta_{c}}{\beta_{d}})\right]}^{2}}{{\left[1+{\left(\Omega \lambda \right)}^{2}\right]}^{2}}$$

(4) $$\beta_{c}=\frac{\sigma_{p}-\sigma_{f}}{\sigma_{p}+2\sigma_{f}},\beta_{d}=\frac{\varepsilon_{p}-\varepsilon_{f}}{\varepsilon_{p}+2\varepsilon_{f}},\lambda =\varepsilon_{0}\frac{\varepsilon_{p}+2\varepsilon_{f}}{\sigma_{p}+2\sigma_{f}}$$

(5) $$\Omega =\frac{\dot{\gamma }}{2},\sigma_{f}=10^{-20\;}S/m$$

where *p*_0_ is the electric dipole moment, d is the diameter of the PANI nanospheres, *β*_eff_ is the dielectric mismatch coefficient, *β*_c_ and *β*_d_ are the low-frequency and high-frequency limits of the Clausius–Mossotti coefficient, respectively, *ε*_0_ is the vacuum dielectric constant, *ε*_p_ is the dielectric constant of the PANI nanospheres, Ω is the angular velocity, $\dot{\gamma }$ is the shear rate, *σ*_p_ is the conductivity of the PANI nanospheres, *λ* is the relaxation time, *ε*_f_ is the dielectric constant of the dispersing medium, and *σ*_f_ is the conductivity of the dispersing medium.

#### 3.1.2. Electric Dipole Moment of Nanofibers

The nanofiber is composed of five small balls of the same volume and can be regarded as a rigid body. The nanofibers have a total volume fraction of 10 vol% and there are 191 nanofibers in the simulation and the number of balls is 955, while the size of a small ball is $d^{\prime }=5.848\times 10^{-7}\;m$. The ball generates an induced electric dipole moment $p_{0}^{\prime }$ subjected to a certain electric field strength *E*_0_ along the *y*-axis, which is expressed as follows:

(6) $${\vec{p}}_{0}^{\prime }=\frac{1}{2}\pi \varepsilon_{0}\varepsilon_{f}d^{\prime 3}\beta_{\mathrm{eff}}{\vec{E}}_{0}$$

Similar results can be obtained for the electric dipole moments $p^{\prime }$ of the stabilized nanofibers:

(7) $${\vec{p}}^{\prime }=\frac{p_{0}^{\prime }}{1+{\left(\Omega \lambda \right)}^{2}}{\vec{e}}_{y}+\frac{\Omega \lambda p_{0}^{\prime }}{1+{\left(\Omega \lambda \right)}^{2}}{\vec{e}}_{x}$$

#### 3.1.3. Electric Dipole Moment of Nanoplates

The nanoplate is composed of sixteen small balls of the same volume and can be regarded as a rigid body. The nanoplates have a total volume fraction of 10 vol% and there are 191 nanoplates in the simulation and the number of small balls is 3056, while the size of a small ball is $d^{\prime \prime }=3.9686\times 10^{-7}\;m$. The ball generates an induced electric dipole moment $p_{0}^{\prime \prime }$ subjected to a certain electric field strength *E*_0_ along the *y*-axis, which is expressed as follows:

(8) $${\vec{p}}_{0}^{\prime \prime }=\frac{1}{2}\pi \varepsilon_{0}\varepsilon_{f}d^{\prime \prime 3}\beta_{\mathrm{eff}}{\vec{E}}_{0}$$

Similar results can be obtained for the electric dipole moments of the stabilized nanoplates $p^{\prime \prime }$:

(9) $${\vec{p}}^{\prime \prime }=\frac{p_{0}^{\prime \prime }}{1+{\left(\Omega \lambda \right)}^{2}}{\vec{e}}_{y}+\frac{\Omega \lambda p_{0}^{\prime \prime }}{1+{\left(\Omega \lambda \right)}^{2}}{\vec{e}}_{x}$$

### 3.2. Definition of Force Field

The basic principles of the MD-SRD method used here have been widely explained elsewhere [53,54,55], so we only present a brief description here. In stochastic rotation dynamics (SRD), the solvent is simulated using *N* point-like particles arranged in regular lattice cubic units, with no limit on the number of particles per unit. There are two main processes of system evolution: flow process and collision process. During the flow process, each particle’s coordinates change as its displacement changes over a time step. The collision process is simulated by synchronously randomly rotating the relative velocities of particles within each unit. In this work, the interaction between individual matrix particles is analyzed according to the SRD method. For the dynamics between dispersed-phase PANI particles-dispersing medium particles, we use the 12-6 Lennard–Jones (L-J)/cut potential function [53].

(10) $$U_{\mathrm{pf}}=\left\{\begin{array}{c} 4u_{\mathrm{pf}}[{\left(\frac{d_{\mathrm{pf}}}{r}\right)}^{12}-{\left(\frac{d_{\mathrm{pf}}}{r}\right)}^{6}]\\ 0 \end{array}\right.\begin{array}{c} \left(r\leq r_{c}\right)\\ \left(r>r_{c}\right) \end{array}$$

where *u*_pf_ represents the depth of the potential well, *d*_pf_ represents the distance when the potential function is zero between dispersed-phase PANI particles and dispersing medium particles, and *r* represents the distance from the dispersed-phase PANI particle center to the dispersing medium particle center.

The electric dipole moment interaction of dispersed-phase particles is determined according to the LJ/cut/dipole/cut potential after applying the electric field.

(11) $$U_{\mathrm{pp}}=\frac{1}{r^{3}}({\vec{p}}_{i}\cdot {\vec{p}}_{j})-\frac{1}{r^{5}}({\vec{p}}_{i}\cdot \vec{r})({\vec{p}}_{j}\cdot \vec{r})$$

(12) $$U_{\mathrm{pp}-\mathrm{LJ}}=\left\{\begin{array}{c} 4u_{\mathrm{pp}}[{\left(\frac{d_{\mathrm{pp}}}{r}\right)}^{12}-{\left(\frac{d_{\mathrm{pp}}}{r}\right)}^{6}]\\ 0 \end{array}\right.\begin{array}{c} \left(r\leq r_{c}\right)\\ \left(r>r_{c}\right) \end{array}$$

where *u*_pp_ represents the depth of the potential well, *d*_pp_ represents the distance when the potential function is zero between dispersed-phase PANI particles, and *r* represents the *r* represents the distance from center to center between dispersed-phase PANI particles.

### 3.3. Performance Characterization

The shear flow field is generated using the box deformation method. We divide the fluid into 10 layers from the top to the bottom of the box. By simulating the velocity of each layer, the velocity profile of the system is acquired.

(13) $$\tau =\eta \frac{dv}{dy}=\eta \dot{\gamma }$$

(14) $$P_{xy}=-\eta \frac{dv}{dy}=-\eta \dot{\gamma }$$

where *τ* is the shear stress and *η* is the viscosity.

Through simulation, we obtain the momentum transfer in the process and the velocity distribution of this system, and then the viscosity of this system can be determined using Equation (14). The obtained viscosity value is substituted into Formula (13), and the shear stress and shear rate curves are ultimately derived.

### 3.4. Simulation Details

All calculations and simulations in this paper are performed using the software Large-scale Atomic/Molecular Massively Parallel Simulator (LAMMPS, 23 October 2017 version). LAMMPS is commonly used by researchers to model a variety of solid metal materials, biomacromolecules, polymers, semiconductor materials, gases, and various coarse-grained particles, with a wide range of applications at the atomic, mesoscopic, and even continuous scales. Through LAMMPS calculation, the position, velocity, force, and various thermodynamic and statistical mechanics-related quantities of each particle in the system can be obtained. In order to take full advantage of computational simulation, that is, to directly observe structural changes, movements, and forces at the microscopic scale, it is necessary to visualize this information [56,57,58,59]. The visualization software used in this article is OVITO-2.9.0.

Three-dimensional models are used for the simulation. The PANI particles are initially arranged in a uniform manner within the simulation box, followed by the addition of matrix particles into the same box. We eliminate overlapping solvent and nanoparticle particles to ensure a realistic initial system, then run 5 × 10^6^ steps for even particle distribution. Next, we apply a certain shear rate along the *x*-axis and a certain electric field strength *E*_0_ along the *y*-axis. Finally, the system runs 10^8^ steps to collect viscosity values.

In the simulation, all parameters utilized are expressed in LJ units. For LJ units, all quantities are unitless. The units of *m*_0_, *d*_0_ and *u*_0_ represent the mass, distance, and energy, respectively. The above three physical quantities are considered basic quantities. LAMMPS sets the fundamental quantities as equal to 1, combined with the experimental requirements, *d*_0_ represents an actual distance of 1 μm, the density of PANI is 1.3 × 10^3^ kg/m^3^, and *m*_0_ is set as 6.8068 × 10^−16^ kg. All masses, distances, and energies in the paper are multiples of these fundamental values. The particle volume fraction is 10 vol%. The dispersing medium mass density *ρ*_f_ is 960 kg/m^3^, and the mass *m*_f_ is identified as 0.01*m*_0_. The above three basic quantities are able to represent additional physical quantities; for example, *t*_0_ represents a time unit, $t_{0}={\left(d_{0}^{2}m_{0}/u_{0}\right)}^{1/2}$. The simulation temperature *T*_0_ is set at 298 K. *u*_0_ = 2.5 *k*_B_*T*_0_. The simulation box has a side length of *Lx* = *Ly* = *Lz* = 10 *d*_0_.

## 4. Results and Discussion

### 4.1. Particle Structure and Morphology

**Figure 4:**
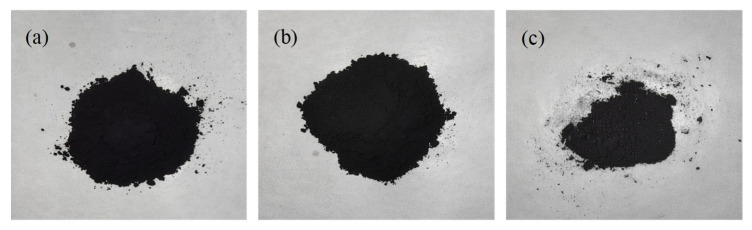
The photographs of the prepared PANI nanoparticles: (**a**) nanosphere; (**b**) nanofiber; (**c**) nanoplate.

**Figure 5:**
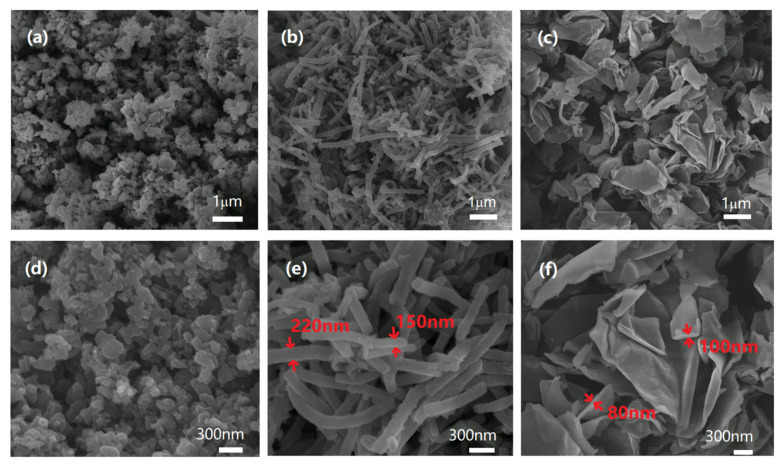
The SEM images of PANI nanoparticles: (**a**,**d**) nanosphere; (**b**,**e**) nanofiber; (**c**,**f**) nanoplate.

Figure 4 shows the photographs of the prepared PANI nanoparticles. These particles are black after dedoping. Figure 5 depicts the SEM image corresponding to the PANI nanospheres, nanofibers, and nanoplates. It can be seen from Figure 5a,d that the PANI synthesized using traditional oxide polymerization in the strong acid solution and with strong stirring comprises aggregated granular particles with a size of about 0.5–1 μm. The PANI synthesized via oxide polymerization under moderately acidic and static conditions, as shown in Figure 5b,e, exhibits a morphology of nanofiber-like particles with diameters varying from approximately 150 to 250 nm and lengths ranging from 1 to 3 μm. These difference in shape occur because aniline reacts with citric acid to form a salt micelle in an aqueous solution and the polymerization of aniline occurs on the surface of micelles to form different nanostructures of PANI. The shape of the micelle depends on the ratio of aniline to citric acid [60,61,62]. When the citric acid concentration is high, the spherical micelle grows and PANI nanospheres are obtained. In the case of a low citric acid concentration, a one-dimensional micelle can easily form and nanofibers are obtained. The PANI produced via oxide polymerization using graphene oxide as a template can be observed in Figure 5c,f as nanoplate-like particles, with a thickness ranging from 80 to 100 nm and a 1–3 μm lateral dimension. The formation of PANI nanoplates occurs due to the presence of a graphene oxide nanosheet as a template.

**Figure 6:**
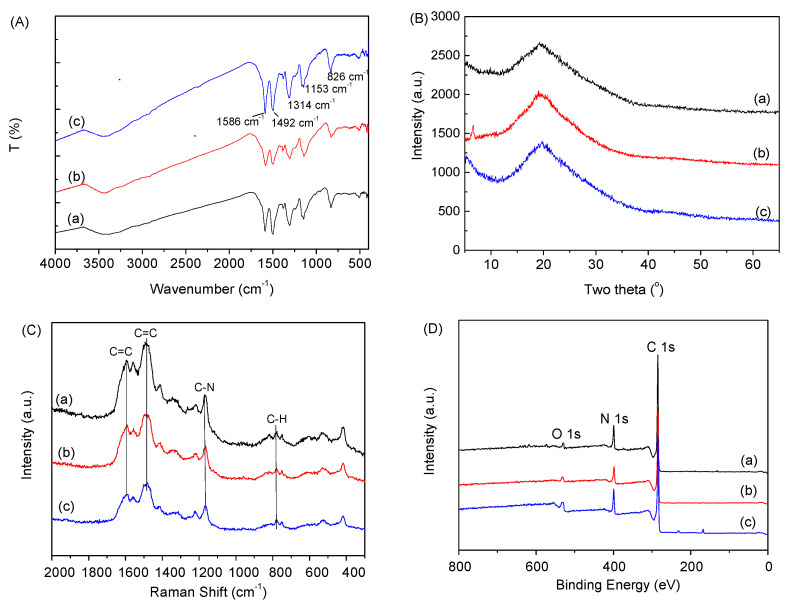
(**A**) The FT-IR spectra, (**B**) XRD pattern, (**C**) Raman spectra, and (**D**) XPS spectra of PANI nanoparticles: (a) nanosphere; (b) nanofiber and (c) nanoplate.

The FTIR spectra of the nanofibers, nanoplates, and nanospheres are depicted in Figure 6A. It can be seen that these PANI nanoparticles have similar chemical groups. The vibrational bands observed at 1586 and 1492 cm^−1^ are ascribed to the stretching vibration of aromatic C=C bonds present in quinone or benzene structures. The band observed near 1314 cm^−1^ matches the elongation vibration of C-N bonds in aromatic secondary amines, whereas the band detected near 1153 cm^−1^ is ascribed to the vibration from N=Q=N (where Q denotes the quinone ring) with electron-like characteristics. On the PANI linear main chain, there is an out-of-plane deformation vibration of the C-H bond in 1, 4-disubstituted benzene detected at 826 cm^−1^. The multiple absorption bands displayed in the region between 3500 and 2800 cm^−1^ primarily match an elongation vibration of N-H bonds originating from secondary amine groups present in the backbone structure of PANI. Figure 6B shows the XRD patterns of PANI nanospheres, nanofibers and nanoplates. It can be seen that the PANI nanoparticles with three morphologies have a wide peak at 20°, which is caused by the period parallel to the polymer chain. It is also found that PANI nanofibers have a small diffraction peak at 6.35°, indicating that PANI nanofibers have a long-range order, which is also consistent with the results of the well-preserved morphology of PANI nanofibers. However, the diffraction peak at 6.35° is not observed in PANI nanospheres and nanoplates, which may be due to the short-range ordering of PANI nanospheres and nanoplates. Figure 6C shows the Raman spectra of PANI nanospheres, nanofibers and nanoplates. It can be seen that the three samples exhibit typical and similar absorption peaks of PANI at 1505 cm^−1^ and 1590 cm^−1^ due to the C=C stretching vibrations on the benzene ring and quinone ring, respectively, at 1167 cm^−1^ due to the C-N^+^ stretching vibration in the semi-quinone ring, and at 780 cm^−1^ due to the C-H bond flexural vibration in the benzene ring and quinone ring. Figure 6D presents the XPS spectrum of the PANI nanospheres, nanofibers and nanoplates. It can be seen that the PANI samples mainly consist of C and N elements. The presence of O is also found, which may be due to the adsorption of pollutants in the air. The content of C and N is similar in the three samples.

We measured the conductance of the three PANI nanoparticles via the pellet method and examined the dielectric spectra of the three PANI ER suspensions. Table 1 lists the electrical conductance values of the particles and the dielectric parameters of the ER suspensions at room temperature. The electrical conductance (*σ*) of PANI nanospheres is 5.6 × 10^−10^ S/cm, that of PANI nanofibers is 6.2 × 10^−10^ S/cm and that of PANI nanoplates is 6.5 × 10^−10^ S/cm. Their conductivities are controlled to be very similar, which will merit ER effect comparison. The three ER suspensions show an interfacial polarization process with a loss peak at 100–120 Hz. The dielectric relaxation time (*λ*) of the PANI nanosphere ER suspension is 1.7 × 10^−3^ s, that of the PANI nanofiber ER suspension is 1.3 × 10^−3^ s and that of the PANI nanoplate ER suspension is 1.3 × 10^−3^ s. The relative dielectric constant of the high-frequency limits (*ε*′_∞_) of the PANI nanosphere ER suspension is 3.05 and the dielectric constant difference (Δ*ε*′ = *ε*′_0_ − *ε*′_∞_ reflects the dielectric intensity of the interface polarization, while *ε*′_0_ and *ε*′_∞_ represent relative dielectric constant with low and high frequencies, respectively) is 2.20, the *ε*′_∞_ of the PANI nanofiber ER suspension is 3.02 and the Δ*ε*′ is 2.20, while the *ε*′_∞_ of PANI nanoplate ER suspension is 3.07 and the Δ*ε*′ is 2.06. The dielectric parameters of the three PANI ER suspensions exhibit remarkable similarity. In particular, although there is a graphene oxide sheet as a supporting core for the formation of the PANI nanoplates, the graphene oxide core does not influence the conductivity and dielectric properties. This may be because the graphene oxide core is very thin, its content is almost ignored, and it is electrically insulating.

Based on the above characterization, three shapes of PANI nanoparticles were prepared. They possess nearly identical chemical structures and electrical parameters. Therefore, the influence of chemical and physical properties on the ER effect can be excluded, with particle morphology being the primary factor influencing the ER effect. After drying, the PANI nanoparticles are dispersed in silicone oil to form ER suspensions. Because the PANI nanoparticles have a good hydrophobic nature and silicone oil has low surface tension, meaning that the PANI nanoparticles can very easily disperse in silicone oil. In addition, the density of organic PANI is low and the particles are relatively stable in the ER suspension. We investigate the ER effect using rheological tests below.

### 4.2. Impact of Nanoparticle Morphology on ER Effect

**Figure 7:**
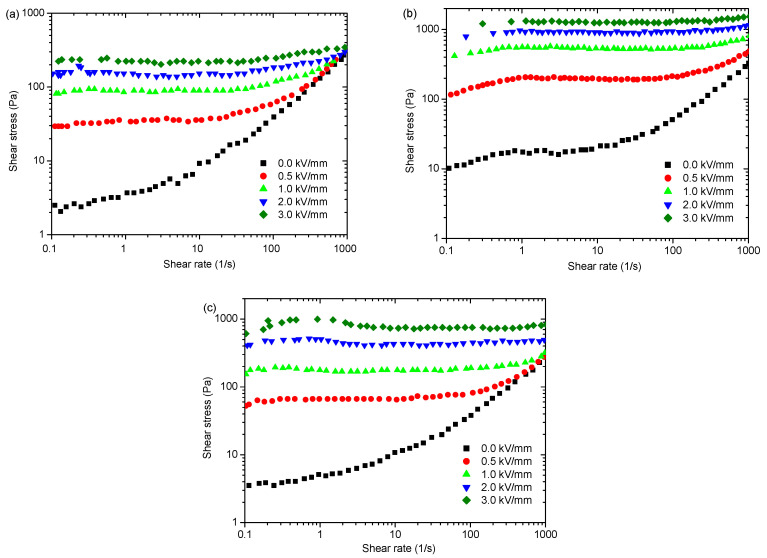
Experimental results of rheological behavior of PANI ER suspensions: (**a**) nanosphere; (**b**) nanofiber; (**c**) nanoplate.

The rheological curves of ER suspensions at the same particle concentration are depicted in Figure 7a–c. The viscosity of the ER suspensions is low without the electric field, but the morphology affects the flow. Figure 7a–c demonstrate that the nanofiber ER suspension has a yield stress of 10 Pa without an electric field. The increase in shear rate leads to significant shear thinning. The nanoplate ER suspension has a small zero-electric field yield stress of 4.5 Pa, and the shear thinning phenomenon becomes weak. However, the nanosphere ER suspension has an even smaller yield stress of 2.5 Pa at zero electric field and the shear thinning phenomenon is further weakened. This indicates that the nanofibers can easily form a three-dimensional network structure in the fluid without applying an electric field, and thus experience yield stress. The three-dimensional network construction of the nanoplate ER suspension becomes weak. The formation of three-dimensional network structures by PANI nanospheres is lacking. When subject to the electric field, the shear stresses of the three ER suspensions increase sharply, and they exhibit obviously larger yield stress. This phenomenon primarily arises from interparticle interactions induced by particle polarization, which causes particles to form chain structures across electrodes [1]. With a rise in shear rate, the shear stresses of ER suspensions under an electric field remain consistently stable, which indicates that the interparticle interaction induced through an electric field can balance with the hydrodynamic force induced through shear flow. However, by comparing the yield stress values among ER suspensions with varying particle morphologies, we can see obvious differences.

**Figure 8:**
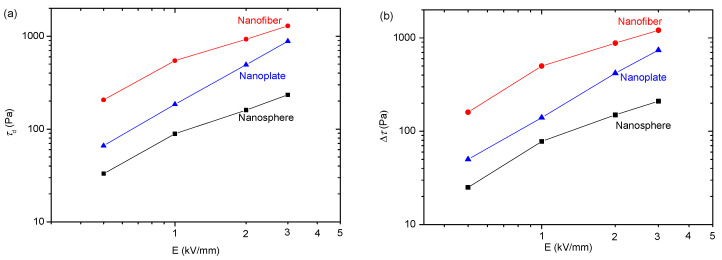
The experimental results of field intensity dependence of (**a**) dynamic yield stresses (*τ*_d_) and (**b**) electric field-induced shear stress increment (∆*τ*) of PANI ER suspensions.

To show the differences more clearly, we extracted dynamic yield stresses (*τ*_d_) utilizing the Bingham function to fit the rheological curves in Figure 7. The yield stress’ dependence on electric field intensity is depicted in Figure 8a. The yield stresses increase as electric field strength rises. When considering spherical particle morphology, the corresponding yield stress value appears to be minimal, while for nanoplates, it falls between these two extremes, and nanofibers exhibit the highest values. We also extracted the electric field-induced shear stress increment (∆*τ* = *τ*_E_ − *τ*_0_, where *τ*_E_ and *τ*_0_ represent the shear stress with and without an electric field, respectively) for three ER suspensions at 1000 s^−1^ and plotted its change with an electric field strength in Figure 8b. Obviously, ∆*τ* is minimal for spherical particles, that of nanofibers is the largest and that of nanoplates is between the two. This finding aligns with the results obtained for yield stresses. These results reflect that the particle morphology significantly influences the ER effect. Moreover, the ER effect is enhanced as the anisotropy of particle morphology increases. A similar result is also obtained in the magnetorheological suspensions studied by Bell et al. [63], Lopez-Lopez et al. [64,65,66,67], and Juan de Vicente et al. [68].

**Figure 9:**
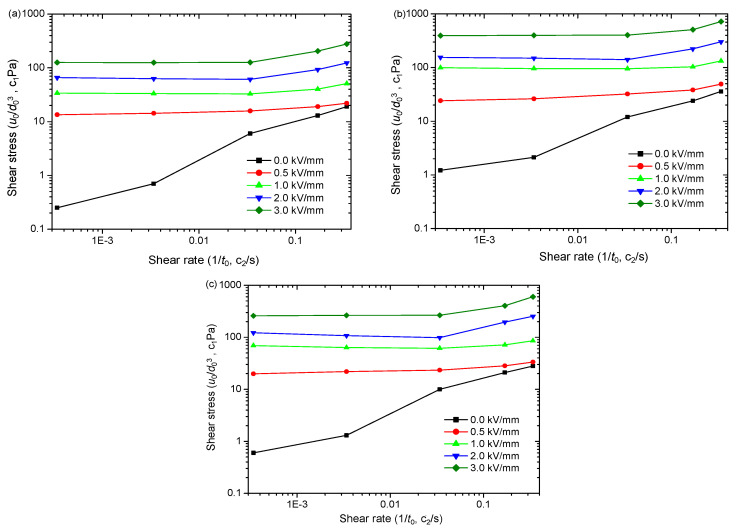
The simulation results of rheological curves of PANI ER suspensions: (**a**) nanosphere; (**b**) nanofiber; (**c**) nanoplate (c_1_ and c_2_ are constants).

We further conduct simulations on the rheological behaviors of PANI ER suspensions with three different morphologies via the MD-SRD method, and Figure 9 shows the simulated results. The three ER suspensions exhibit low viscosity under zero electric field, and the shear thinning is obvious as the shear rate increases. The nanofiber ER suspension clearly shows the highest yield stress without any electric field, followed by the nanoplate ER suspension, with a relatively lower yield stress, and finally the nanosphere ER suspension, with the lowest yield stress. This aligns well with the experimental findings exhibited in Figure 7. This can be attributed to the fact that the elongated nanofibers and nanoplates can easily form a complex three-dimensional network structure compared to nanospheres without an electric field, as shown in Figure 2. Thus, when nanofiber and nanoplate ER suspensions are subjected to shear stress, the solid friction between nanofibers and nanoplates in contact with each other can induce yield stress [63]. Under electric fields, regardless of the morphology, the shear stress values exhibit a rise as electric field intensity increases, as shown in Figure 9a–c. However, by comparing the yield stress values of ER suspensions with varying nanoparticle morphologies, we can also see obvious differences.

**Figure 10:**
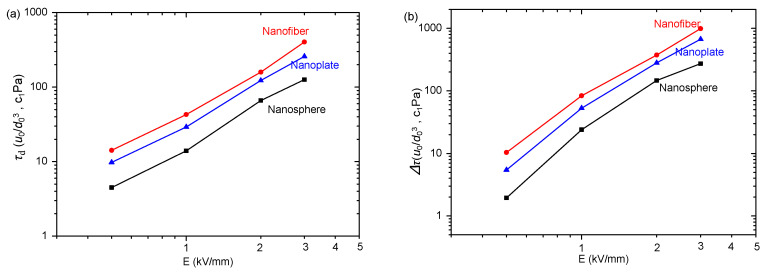
The simulation results of field intensity dependence of (**a**) dynamic yield stresses (*τ*_d_) and (**b**) electric field-induced shear stress increment (∆*τ*) of PANI ER suspensions. (c_1_ is a constant).

Similarly, we extracted the dynamic yield stress (*τ*_d_) and electric field-induced shear stress increment (∆*τ*) from Figure 9 and depict the results as the function for electric field intensity in Figure 10a,b. It can be observed that the nanosphere ER suspension exhibits the lowest yield stress and shear stress increment, while the corresponding values for the nanoplate ER suspension are slightly higher, and those of the nanofiber ER suspension are the largest. This is consistent with the experimental results in Figure 8. These results also demonstrate that particle morphology significantly influences the ER effect and that an increase in particle anisotropy leads to an enhancement of the ER effect. In previous reports, some studies have compared the ER effect of spherical and fiber-like nanoparticles [17,18,19,20,21,22,23,24,25,26,27], and some have compared the ER effect of spherical and plate-like particles, and they found that anisotropic particles have a better ER effect [35,36,37,38,39,40,41,42]. Up to now, however, no one has systematically compared the three morphologies (sphere, fiber, plate) of ER particles. The present study shows that the ER effect is enhanced as the particle anisotropy increases by comparing the three morphologies (sphere, fiber, plate) of ER particles.

**Figure 11:**
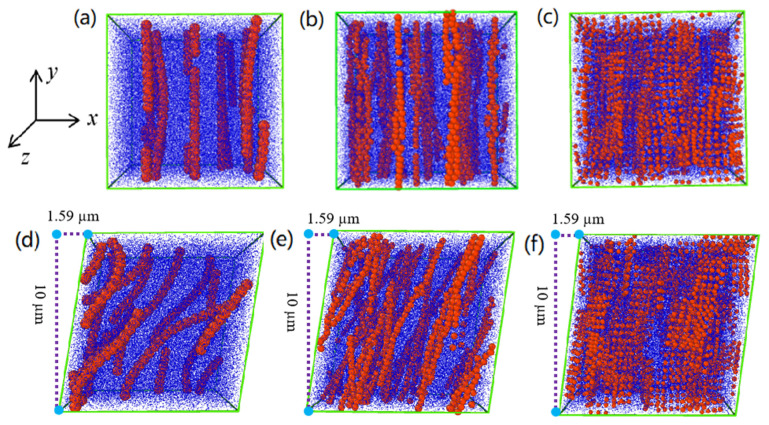
Microstructure of PANI-based ER suspensions at 3 kV/mm without shear stress (**a**–**c**) and with shear stress (**d**–**f**): (**a**,**d**) nanosphere; (**b**,**e**) nanofiber; (**c**,**f**) nanoplate.

To analyze how the ER effect is affected by the particle morphology, we further proceed with a three-dimensional visual simulation. In Figure 11, we conduct a comparative analysis of the evolution of particle morphologies in ER suspensions with distinct microstructures. It is obvious from Figure 11a–c that under an electric field, the particles undergo polarization and mutual attraction to produce chain structures in the direction of the electric field. The sphere-like particles can easily form single chains containing defects, but the fiber-like particles connect together through their sides or overlap, forming a complex chain structure. The plate-like particles also form a complex chain structure with different lateral orientations. This complex chain structure is considered to exhibit greater stability compared to the simple chain structure generated using spherical particles [20,21,64,65,66,67,68]. When shear stress is applied, as shown in Figure 11d, the chain structure of the spherical particles inclines and breaks into short chains. Meanwhile, as shown in Figure 11f, the chain structure formed from plate-shaped particles inclines and also breaks when shear stress is applied. As shown in Figure 11e, when shear stress is applied the chain structure formed from fiber-shaped particles inclines but forms a more complex dendritic network structure. This complex dendritic network structure can prevent the particles from sliding together and suppress chain structure fracture, thereby improving the ER effect [21,64]. Therefore, based on the three-dimensional visual simulation, we can approximately conclude that the ER effect is likely enhanced due to the increased stability of the ER chain structure as particle anisotropy increases. Moreover, the electrostatic force between elongated particles is considered to be larger than that between spherical particles due to the enhanced long-axis polarizability, which also contributes to the increase in the ER effect as particle anisotropy increases [19,31].

## 5. Conclusions

In this paper, three shapes of PANI nanoparticles, including nanospheres, nanofibers, and nanoplates, have been prepared. They possess nearly identical chemical structures and electrical parameters. The impact of nanoparticle morphology on the ER effect has been studied through rheological experiments and molecular dynamics simulations. The experimental results demonstrate that, under identical conditions, the enhancement of the ER effect is positively correlated with an increase in particle morphology anisotropy. PANI nanofibers exhibit the highest ER effect, followed by nanoplates, while PANI nanospheres display the smallest ER effect. This observation is consistent with the simulation results. Three-dimensional visual simulations reveal that the enhanced ER effect associated with increased particle anisotropy can be attributed to the improved stability of the ER chain structure. Based on this result, the synthesis of other ER materials with elongated morphology can be one direction of future research into high-performance ER materials. In addition, although the simulation result obtained at the micro scale is generally similar to that obtained in the actual experiment, there are still many differences. In the future, a particle model closer to the actual morphologies should be established for ER effect simulation.

## Figures and Tables

**Table 1:** The conductance of PANI nanoparticles and dielectric parameters of PANI ER suspensions with different particle morphologies (*ϕ* = 10 vol%).

Sample	*ε*′_∞_	Δ*ε*′	*λ* (s)	*σ* (S/cm)
nanosphere	3.05	2.20	1.7 × 10^−3^	5.6 × 10^−10^
nanofiber	3.02	2.21	1.3 × 10^−3^	6.2 × 10^−10^
nanoplate	3.07	2.06	1.3 × 10^−3^	6.5 × 10^−10^

## Data Availability

Data will be made available upon request.
